# Comparison of covariate adjustment methods using space-time scan statistics for food animal syndromic surveillance

**DOI:** 10.1186/1746-6148-9-231

**Published:** 2013-11-18

**Authors:** Gillian D Alton, David L Pearl, Ken G Bateman, Bruce McNab, Olaf Berke

**Affiliations:** 1 Department of Population Medicine, Ontario Veterinary College, University of Guelph, Guelph, ON N1G 2W1, Canada; 2 Ontario Ministry of Agriculture & Food, Guelph, ON N1G 4Y2, Canada; 3 Department of Mathematics and Statistics, University of Guelph, Guelph, ON N1G 2W1, Canada

**Keywords:** Scan statistic, Syndromic surveillance, Abattoir, Condemnations

## Abstract

**Background:**

Abattoir condemnation data show promise as a rich source of data for syndromic surveillance of both animal and zoonotic diseases. However, inherent characteristics of abattoir condemnation data can bias results from space-time cluster detection methods for disease surveillance, and may need to be accounted for using various adjustment methods. The objective of this study was to compare the space-time scan statistics with different abilities to control for covariates and to assess their suitability for food animal syndromic surveillance. Four space-time scan statistic models were used including: animal class adjusted Poisson, space-time permutation, multi-level model adjusted Poisson, and a weighted normal scan statistic using model residuals. The scan statistics were applied to monthly bovine pneumonic lung and “parasitic liver” condemnation data from Ontario provincial abattoirs from 2001–2007.

**Results:**

The number and space-time characteristics of identified clusters often varied between space-time scan tests for both “parasitic liver” and pneumonic lung condemnation data. While there were some similarities between isolated clusters in space, time and/or space-time, overall the results from space-time scan statistics differed substantially depending on the covariate adjustment approach used.

**Conclusions:**

Variability in results among methods suggests that caution should be used in selecting space-time scan methods for abattoir surveillance. Furthermore, validation of different approaches with simulated or real outbreaks is required before conclusive decisions can be made concerning the best approach for conducting surveillance with these data.

## Background

With the development and availability of geographic information systems (GIS), there has been an increasing trend in human and animal disease surveillance towards capturing both temporal and spatial data for health and disease outcomes. Spatio-temporal scan statistics are one of the most widely used methodologies [1] for surveillance and have been shown to be useful for surveillance and outbreak detection in both human and animal health applications [2-7]. Space-time scan statistics are one type of spatio-temporal surveillance method which uses a cylindrical scanning window to scan spatially by varying the size of the cylinder radius and scan temporally by varying the height of the cylinder. Statistical significance of the cluster is determined by Monte Carlo based simulations to adjust for multiple hypothesis testing. Analysis can be conducted both retrospectively, as well as prospectively, making it suitable for disease surveillance [8].

Syndromic surveillance is the amalgamation of signs/symptoms using data from non-traditional sources [9]. The sign/symptom groupings are loosely designated as ‘syndromes’ , and are used to track disease trends in populations and signal a possible outbreak that warrants further investigation [9]. Historically, syndromic surveillance has primarily been applied to human health data [10-12]. However, in recent years there has been a growing trend towards the application of these methods for animal health surveillance data [13-17]. Abattoir condemnation data are a rich source of information for syndromic surveillance, and have the potential to provide early warning of emerging animal and zoonotic disease but have been under-utilized in the past. Ontario provincial abattoir data are particularly advantageous for syndromic surveillance and the application of spatio-temporal methods, as they represent a fairly local picture of animal health events, with cattle being shipped to abattoirs originating from farms less than 100 km away [17].

Scan statistics identify the approximate locations of disease clusters in space and time, and make use of a variety of statistical models [1,2], making them useful for a variety of data. However, space-time scan statistics and current available software do have some limitations and assumptions, which may be violated by the inherent characteristics of provincial abattoir data. For example, while the space-time scan statistic has the ability to control for covariates, at this time, this is only applicable to categorical variables, thus limiting the type of variables one can control for in the analysis [1]. The space-time permutation model inherently corrects for purely spatial and purely temporal clusters, however, the expected rates of disease are dependent on a relatively stable background population [18]. While this is generally true for human populations and periods of a few years, this is generally not the case with abattoir data, where animal population sizes can vary by season.

In recent years, model-based approaches have emerged to account for covariates such as age, gender, and seasonality in expected rates of disease, in response to the limited ability of space-time scan statistic software to include these covariate data [1]. Statistical modeling allows for adjustment of disease risk for both categorical and continuous variables in space and time. By combining both methods, surveillance researchers have the ability to account for relevant covariates, while locating clusters in space and time. A study by Kleinman et al. [6], used this approach by conducting model-adjusted space-time scan tests for syndromic surveillance of lower respiratory complaints in a human health care setting. The study controlled for non-disease factors such as day of week, month, and holidays and found that the number of false alarms could be reduced by removing the “noise” of predictable covariates. However, this method has not been applied to animal condemnation data for disease surveillance. Previous studies by Alton et al. and Thomas et al. found that various seasonal, secular, disease and abattoir characteristic factors were associated with condemnation rates in Ontario provincial abattoirs; they stressed that these might be accounted for in the application of quantitative space-time cluster detection methods for disease surveillance involving abattoir data [17-19]. This study also highlights the importance of thinking beyond the typical age and sex covariate adjustment and controlling for disease and non-disease factors such as animal throughput at the abattoir and sales price of the animal class which may have a considerable impact on the results, particularly for abattoir condemnation data.

Due to the variety of methods available for covariate-adjustment in cluster detection, and their varying level of complexity in terms of analysis, a comparison study of the space-time scan statistic on four different approaches at controlling for covariates was used. If similarities in results were found between multiple approaches, then the most parsimonious model could be recommended. Four covariate adjusted scan tests were compared to results from the unadjusted space-time scan test including: 1) categorical variable adjustment which stratifies on the covariate variable of interest within the space-time scan statistic, 2) space-time permutation model which uses only case data and inherently controls for purely spatial and purely temporal clusters, 3) multi-level model adjusted approach which allows for adjustment of both categorical and continuous variables, and 4) multi-level model residual-adjusted approach which uses the standardized residuals from the above motioned multi-level model to control for covariates.

The objective of this study was to demonstrate four commonly used space-time scan statistic approaches with different abilities to control for covariates that animal health surveillance workers might consider when using statistical methods to identify outbreaks of disease using abattoir condemnation data and assess their suitability for food animal syndromic surveillance involving Ontario provincial abattoir condemnation data.

## Methods

### Data source and variables

Data regarding bovine “parasitic liver” and pneumonic lung condemnations were extracted from the Food Safety Decision Support System (FSDSS) database maintained by the Ontario Ministry of Agriculture, Food and Rural Affairs (OMAFRA). The database contains information regarding the number and reason for daily portion condemnations in provincially inspected abattoirs in Ontario. These portion condemnation categories were selected for this analysis as an example dataset, as they were among the most frequently reported portion condemnations by provincial inspectors during the study period [19]. Additionally, as bovine livers are an edible portion, these data may represent a potential food safety and/or quality concern. “Parasitic liver” is an inspection term used to label bovine livers considered unfit for human consumption, due to lesions such as necrosis, fibrosis, cirrhosis, atrophy, telangiectasia, and adhesions. Although the term “parasitic liver” suggests truly parasitic infections such as fascioliasis, the term covers non-parasitic conditions as well (personal communication Abdul Rehmtulla, DVM, OMAFRA, Stone Road, Guelph, Ontario). Pneumonic lung condemnation refers to bovine lungs which were condemned for lesions indicative of a previous localized and resolved antero-ventral pneumonia infection.

Data were extracted from the database for cattle animal classes: bulls, calves, cows, heifers and steers from January 1, 2001 – December 31, 2007. Data from 45,148 bulls were excluded from subsequent analyses due to missing data and inconsistencies in the use of this classification. Missing geographical coordinates for 54 abattoirs (26%) were approximated using postal codes (76%) and/or addresses (24%) with the address geocoding software GeoPinpoint Suite 6.4 (DMTI Spatial Inc., Markham, Ontario, Canada).

### Space-time scan statistic

The space-time scan statistic was used to identify abattoirs with high and low “parasitic liver” and pneumonic lung condemnation rates in space-time using SaTScan v8.0 (Kulldorff M. and Information Management Services Inc., 2009.), and were visualized on maps using ArcGIS 9.2 (ESRI, Redlands, California, USA). Four different approaches to control for confounding variables were compared to each other and an unadjusted Poisson scan statistic including: (1) animal class adjusted Poisson scan statistic, (2) space-time permutation, (3) multi-level model adjusted Poisson scan statistic, and (4) a weighted normal scan statistic using model residuals. For all scan tests, latitude and longitude coordinates for each abattoir, and premise identification number were used to create the coordinates file. A maximum spatial cluster size of 50% of the population at risk and maximum temporal cluster size of 50% of the study period were used. For all scan tests, 9999 Monte Carlo replications were used to estimate the significance levels of the clusters. For all analyses, the most likely (based on the size of the log-likelihood ratio), non-overlapping in space-time, statistically significant (α = 0.05) clusters are presented. Secondary clusters were set to allow some overlap as long as the secondary cluster and a previously reported cluster did not both contain each other’s centroid. By allowing some overlap, we were able to identify space-time clusters that overlapped in space, but not time. Only the most likely non-overlapping clusters were reported to simplify the presentation of the results. All the tests were run as two-sided tests scanning for both high and low levels of disease to identify disease clusters as well as abattoirs with unusually low condemnation rates.

For the unadjusted scan statistic, monthly raw counts of “parasitic liver”/pneumonic lung condemnations and monthly number of cattle slaughtered were used to create the case and population files respectively using a Poisson distribution. In the animal-adjusted scan statistic, monthly raw counts of “parasitic liver”/pneumonic lung condemnations and monthly number of cattle slaughtered were used to create the case and population files respectively using a Poisson distribution. Cattle animal class (e.g., calves, cows, heifers, steers) were adjusted with the space-time scan statistic by stratifying on the variable within the case file. For the space-time permutation model, raw case counts of “parasitic liver”/pneumonic lung condemnations were used. For the model-adjusted scan test, a multi-level model was previously created to identify economic, seasonal and abattoir processing capacity characteristics associated with “parasitic liver” and pneumonic lung condemnation rates. The model identified year, season, animal class, audit rating and region to be statistically associated with “parasitic liver” condemnation rates [19]. For the pneumonic lung condemnation rates year, season, animal class, region, audit rating, number of cattle processed per year, and number of weeks abattoirs processed cattle were found to have a statistically significant association [19]. For the model-adjusted scan statistic, standardized morbidity ratios were used in the space-time scan statistic based on the observed number of condemnations and the model predicted counts. For the model residual scan test, the observation level standardized residuals from the multi-level model were analyzed. A Poisson model was used for the model-adjusted scan test and a weighted normal model was used for the scan test using the multi-level model residuals (at the level of the observation). The normal model assumes that the normal variable (i.e., standardized residual) is independent and identically distributed under the null hypothesis and therefore has the same variance. Since the population at abattoirs changes greatly, the varying sample size at each abattoir will cause the variance to be different for different abattoirs, thus a weighted normal model was used to take into account the uncertainty of the observed rate [20]. The total number of cattle slaughtered at each abattoir at each time period was used to account for the variability.

## Results

A total of 211 provincially-inspected abattoirs, slaughtering a total of 1,155,535 cattle from 2001–2007 were included in this study. Provincially-inspected abattoirs can be found throughout Ontario; however, over 80% of abattoirs processing cattle are located in Southern, Western and Central Ontario regions. “Parasitic liver” and pneumonic lungs condemnations were among the most frequently condemned portions and accounted for approximately 18% and 9% of total condemned portions for the study period, respectively. A complete description of these data and abattoir locations can be found in Alton et al. [19].

### “Parasitic liver” data

**Figure 1:**
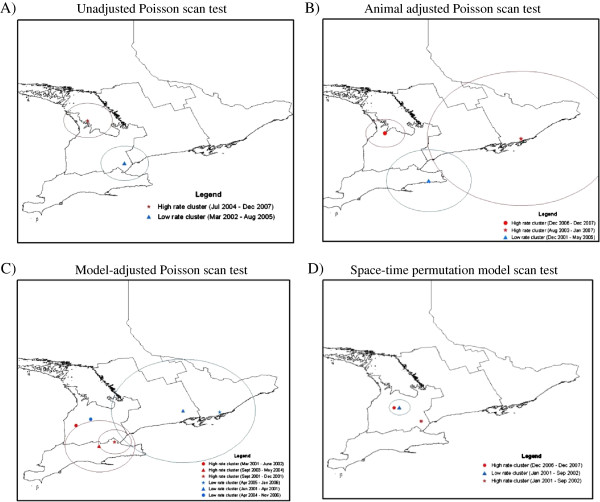
Results of space-time scan statistic using “parasitic liver” portion condemnation data from Ontario provincial abattoirs 2001–2007 using four approaches for covariate adjustment compared to the unadjusted data including: A) Unadjusted Poisson space-time scan test, B) Animal-adjusted Poisson space-time scan test, C) Model-adjusted space-time scan test and D) Space-time permutation scan test.

Results of the unadjusted Poisson space-time scan statistic identified 1 high rate cluster from July 2004 – December 2007, and 1 low rate cluster from March 2002 – August 2005 (Figure 1A and Table 1a). Results of the animal class adjusted space-time scan statistic identified 2 high rate clusters from August 2003 – January 2007 and December 2006 – Dec 2007, and 1 low rate cluster from December 2001 – May 2005 (Figure 1B and Table 1b). The model-adjusted space-time scan identified 3 high rate clusters from September 2001 – December 2001, March 2001 – June 2002, and September 2003 – May 2004, and 3 low rate clusters from January 2001 – April 2001, April 2004 – November 2006 and April 2005 – January 2006 (Figure 1C and Table 1c). The space-time permutation model identified 2 high rate clusters from January 2001 – September 2002 and December 2006 – December 2007, and 1 low rate cluster from January 2001 – September 2002 (Figure 1D and Table 1d). Lastly, the space-time scan test applied to Poisson model residuals did not identify any statistically significant high or low rate clusters.

**Table 1:** Results of the space-time scan statistic using “parasitic liver” condemnation data

**Date of cluster**	**No. of abattoirs in cluster**	**Latitude (°N)**	**Longitude (°W)**	**Radius (Km)**	**No. of cases**	**Relative risk**^**1**^	**P-value**
**a) Unadjusted Poisson space-time scan test**							
Jul 2004 - Dec 2007	16	44.52	80.92	62.45	12907	3.66	< 0.001
Mar 2002 - Aug 2005	26	43.60	79.81	62.52	6385	0.36	< 0.001
**b) Animal-adjusted Poisson space-time scan test**							
Dec 2006 - Dec 2007	14	44.53	80.99	52.98	4973	3.90	< 0.001
Dec 2001 - May 2005	51	42.91	79.50	117.97	8732	0.45	< 0.001
Aug 2003 - Jan 2007	69	44.36	76.35	255.37	13849	1.59	< 0.001
**c) Model-adjusted space-time scan test**							
Mar 2001 - Jun 2002	1	43.90	81.31	0	420	4.91	< 0.001
Sep 2003 - May 2004	71	43.18	80.51	98.22	4576	1.52	< 0.001
Sep 2001 - Dec 2001	16	43.33	79.98	46.01	1335	2.19	< 0.001
Mar 2004 - Nov 2006	2	44.11	80.80	0	2338	0.62	< 0.001
Jan 2001 - Mar 2001	72	44.40	77.61	197.95	496	0.49	< 0.001
Mar 2005 - Jan 2006	1	44.36	76.35	0	56	0.18	< 0.001
**d) Space-time permutation scan test**							
Jan 2001 - Sep 2002	2	43.65	79.86	4	2335	6.76	< 0.001
Jan 2001 - Sep 2003	7	44.13	80.61	29.42	341	0.11	< 0.001
Sec 2006 - Dec 2007	2	44.11	80.80	0	4431	3.06	< 0.001
**e) Space-time scan test of residuals**							
**No statistically significant clusters found**							

^1^The values in this column for the space-time permutation model refer to the observed/expected value.

### Pneumonic lung data

**Figure 2:**
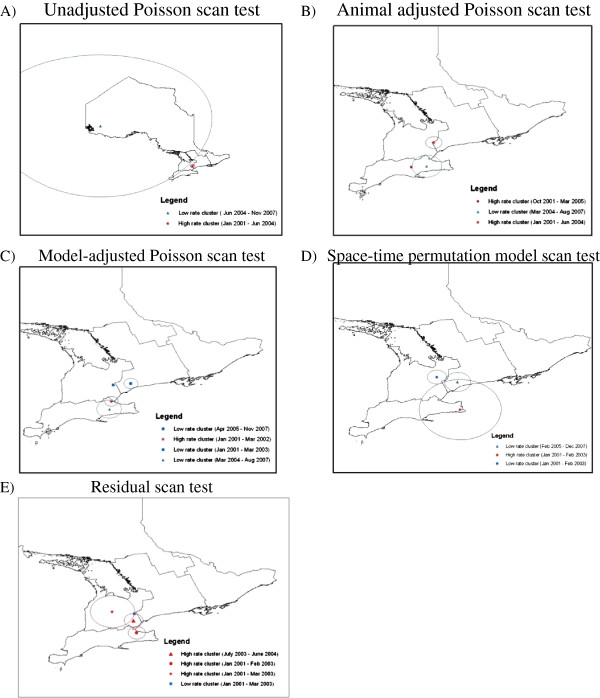
Space-time scan statistic using pneumonic lung condemnation data from Ontario provincial abattoirs 2001–2007 using four approaches for covariate adjustment compared to the unadjusted data including: A) Unadjusted Poisson space-time scan test, B) Animal-adjusted Poisson space-time scan test, C) Model-adjusted space-time scan test and D) Space-time permutation scan test and e) Space-time scan test of residuals.

Results of the unadjusted Poisson space-time scan statistic identified 1 high rate cluster from January 2001 – June 2004, and 1 low rate cluster from June 2004 to November 2007 (Figure 2A and Table 2a). Results of the animal class adjusted space-time scan statistic identified 2 high rate clusters during January 2001 – June 2004 and October 2001 to March 2005, and 1 low rate cluster during March 2004 – August 2007 (Figure 2B and Table 2b). The model-adjusted space-time scan statistic identified 1 high rate cluster during January 2001 – March 2002 and 3 low rate clusters during March 2004 – August 2007, January 2001 – March 2003 and April 2005 – November 2007 (Figure 2C and Table 2c). The space-time permutation model identified 1 high rate cluster during Jan 2001 – February 2003, and 2 low rate clusters during January 2001 – February 2003 and February 2005 – December 2007 (Figure 2D and Table 2d). Lastly, the space-time scan statistic using multi-level model residuals identified 3 high rate clusters during January 2001 – March 2003, July 2003 – June 2004 and January 2001 – February 2003, and 1 low rate cluster during January 2001 – March 2003 (Figure 2E and Table 2e).

**Table 2:** Results of the space-time scan statistic using pneumonic lung condemnation data

**Date of cluster**	**No. of abattoirs in cluster**	**Latitude (°N)**	**Longitude (°W)**	**Radius (Km)**	**No. of cases**	**Relative risk**^**1**^	**P-value**
**a) Unadjusted Poisson space-time scan test**							
Jan 2001 - Jun 2004	5	43.94	79.69	26.60	14896	4.98	< 0.001
Jun 2004 - Nov 2007	63	49.75	92.96	1149.60	54	0.01	< 0.001
**b) Animal-adjusted Poisson space-time scan test**							
Jan 2001 - Jun 2004	5	43.96	79.69	26.60	14896	3.12	< 0.001
Mar 2004 - Aug 2007	21	43.01	79.97	49.09	61	0.01	< 0.001
Oct 2001 - Mar 2005	1	42.98	80.60	0	4466	5.14	< 0.001
**c) Model-adjusted space-time scan test**							
Mar 2004 - Aug 2007	16	43.01	79.97	36.88	60	0.07	< 0.001
Jan 2001 - Mar 2003	1	43.91	79.82	0	1558	0.44	< 0.001
Jan 2001- Mar 2002	4	43.30	79.90	19.98	477	6.06	< 0.001
Apr 2005 - Nov 2007	4	43.96	79.19	20.93	273	0.26	< 0.001
**d) Space-time permutation scan test**							
Jan 2001 - Feb 2003	11	44.14	79.94	28.20	1520	0.31	< 0.001
Jan 2001 - Feb 2003	32	42.95	79.10	121.36	10514	1.55	< 0.001
Feb 2005 - Dec 2007	10	43.96	79.19	38.19	363	0.20	< 0.001
**e) Space-time scan test of residuals**							
Jan 2001 - Mar 2003	1	43.91	79.82	0	110	---	< 0.001
Jan 2001 – Feb 2003	8	43.17	79.73	26.91	370	---	< 0.001
Jul 2003 - Jun 2004	6	43.65	79.86	29.33	109	---	< 0.001
Jan 2001 - Mar 2003	36	43.99	80.67	68.49	2280	---	< 0.001

^1^The values in this column for the space-time permutation model refer to the observed/expected value.

## Discussion

Provincial abattoir condemnation data may be useful for integration into a food animal syndromic surveillance system; however, there are inherent characteristics in the data which can bias the results of quantitative cluster detection methods. The results of this methodological comparison study found differing results depending on the type of covariate adjustment method used. By not considering or accounting for certain covariates, such as non-disease factors (i.e. price, throughput), or selecting an inappropriate statistical model for the data (i.e. space-time permutation model) the subsequent results can lead to very different conclusions. These results suggest caution should be exercised when arbitrarily selecting a space-time scan statistic model for disease surveillance involving these data and highlight the importance of preliminary validation studies using simulated or documented outbreak reports before a standard cluster detection method is adopted by a surveillance system. A proper choice of the method needs to take into account the properties of the sample data (e.g., its distribution, case only vs. case and control data) and the question to be answered by the statistical test (e.g., spatial versus space-time cluster location).

Farm location information is not routinely recorded for provincial abattoir condemnation data. This lack of farm of origin location information for animals being shipped to Ontario provincial abattoirs is a limitation in conducting spatial-temporal cluster analyses. However, a previous study by Alton et al. [17], estimated the distance between the animals’ farm and the abattoir using a subset of cattle, in which a sample was sent for laboratory testing. The authors found cattle were shipped less than 100 km to Ontario provincial abattoirs, and given the spatial scale of Ontario (1,000,000 km^2^), abattoirs are considered to give an appropriate approximation of the disease rates among locally slaughtered cattle. Over 75% of the abattoirs were geo-located by OMAFRA is the FSDSS dataset; approximately 25% had missing co-ordinates and had to be geo-located using addresses and/or postal code information. Of these, 54 abattoirs with missing geo-locations, 76% were geo-located to the centroid of a postal code. However, based on the potential difference between the abattoir location and centroid of its postal code area in relation to the size of the study area, the impact would be negligible.

While there were differences between the four space-time scan statistic approaches particularly for the “parasitic liver” condemnation data, there were some similarities between the different approaches. For example, the “parasitic liver” data had clusters which overlapped in space between the one high rate cluster and one low rate cluster in the unadjusted and animal class adjusted approaches (Figure 1A and B) and again between 2 high rate clusters and one low rate cluster in the model-adjusted and space-time permutation model approaches (Figures 1C and D), however, none of these clusters overlapped in time, and were at least a year apart. In contrast, pneumonic lung data had a similar high rate cluster which overlapped in space and time between the unadjusted and animal adjusted approaches (Figures 2A and B), a low rate cluster between the model-adjusted and space-time permutation approach (Figures 2C and D) and a low rate cluster between the model-adjusted, space-time permutation and residual scan approaches (Figures 2C, D and E). There was also a similar high rate cluster in space and time between the space-time permutation model and the residual scan (Figures 2D and E). While there were isolated clusters which overlapped between different adjustment approaches, overall, the results of the scan statistics depicted very different clusters between the different adjustment methods. The overall differences in results of the covariate adjustment approaches suggest ignoring covariates beyond the animal level may be unwise when using abattoir condemnation data for food animal syndromic surveillance.

While each adjustment approach found differing results, it is also important to consider assumptions of the scan statistic models in relation to the data being used. For example, the space-time permutation model, which uses only case data, is advantageous when population data are missing or difficult to obtain/sample. However, it is likely not appropriate for use with provincial abattoir data, as the model assumes a stable underlying population, which is not the case with these data. It was hypothesized that perhaps the majority of the variation in condemnation rates may be attributed to animal class, and perhaps by simply controlling for this categorical variable (using an adjustment file when employing SaTScan) one would see similar results to that of the model-adjusted approach. However, we found these approaches showed very different results. We suspected that the model-adjusted and model residual adjustment approaches would be the most appropriate for quantitative cluster detection involving provincial abattoir condemnation data, as these methods are able to account for both categorical and continuous variables, making it the most versatile of the adjustment approaches. However, when the event is rare, as in the present example with condemnation rates, the precision of the residuals using the normal model is unstable and may give inaccurate results when applied to the space-time scan statistic [20]. Thus, this approach is not appropriate for the application of provincial abattoir condemnation data unless counts are aggregated to a higher temporal and/or spatial level. The model-adjusted approach using the ratio of observed versus expected condemnations under the Poisson model would be more appropriate when utilizing relatively rare events, as in the case of abattoir condemnation data. However, this approach involves more complex analyses than some of the other approaches. In addition, the current multi-level model includes temporal variables such as, year and season, which are more conducive to retrospective analyses and would be difficult to account for prospectively. Ultimately, to ensure the proper use of these methods, validation of the different approaches with simulated or documented outbreaks needs to be performed in selecting the most appropriate statistical test for these data.

The comparison of the different methods for covariate adjustments highlights the variability in the results for both pneumonic lung and “parasitic liver” portion condemnation data. Overall, the results for both types of portion condemnation data demonstrate that as you increase the detail in the covariate adjustment information, the size of the cluster decreases. A study conducted by Kleinman *et al.*[6] compared the space-time scan statistic using unadjusted and model-adjusted approaches for syndromic surveillance of lower respiratory illness to account for confounding temporal and disease variables, such as day of week, month, holidays and local history of illness. This study found that during influenza season, large space-time clusters were identified almost every weekday by the unadjusted approach compared to the model-adjusted approach, making it unfeasible to investigate all ‘unusual’ events and diminishing the value of the tool for surveillance. This adjustment effect could also be found in the current study, further justifying the need for covariate adjustment with the space-time scan statistic for disease surveillance purposes.

## Conclusions

This study demonstrates the importance of identifying and adjusting for various disease and non-disease factors which may bias the results of cluster detection methods for disease surveillance. When selecting an adjustment method, it is important to consider not only the inherent assumptions in the statistical method, but also these assumptions in relation to the data being utilized. The variability in results stresses that there are a variety of methods currently available for covariate adjustment, and that by simply selecting one such method, without prior research and planning may yield very different and potentially inaccurate results. Background studies such as this, which identify important confounding factors and effectively correct for them may assist in improving the sensitivity and specificity of outbreak detection by controlling for predictable clusters creating false alarms and reducing the amount of time and resources needed for investigation of potential clusters, as well as, identifying outbreaks that would have been normally overlooked in the background “noise” of these data. Ultimately, validation of different approaches with simulated or real outbreaks needs to be performed in selecting the appropriate statistical test for these data for a food animal syndromic surveillance context.

## Competing interests

The authors declare that they have no competing interests.

## Authors’ contributions

GDA was involved in the conception and design of the study, performed the statistical analysis and drafted the manuscript. BM was involved in the acquisition of data, and the drafting and revision of the manuscript for intellectual content. DLP and OB were involved in the conception and design, analysis and interpretation of data, and revising manuscript critically for important intellectual content. KGB was involved in the interpretation of data, and the revising of the manuscript critically for important intellectual content. All authors read and approved the final manuscript.

## References

[B1] Robertson C Nelson TA MacNab YC Lawson AB Review of methods for space–time disease surveillance Spat Spattemporal Epidemiol 2010 1 105 116 10.1016/j.sste.2009.12.001 PMC7185413 22749467

[B2] Pearl DL Louie M Chui L Dore K Grimsrud KM Leedell D Martin SW Michel P Svenson LW McEwen SA The use of outbreak information in the interpretation of clustering of reported cases ofEscherichia coli 0157 in space and time in Alberta, Canada, 2000–2002 Epidemiol Infect 2006 134 699 711 10.1017/S0950268805005741 16388687 PMC2870460

[B3] Odoi A Martin SW Michel P Middleton D Holt J Wilson JB Investigation of clusters of giardiasis using GIS and a spatial scan statistic Int J Health Geogr 2004 3 11 10.1186/1476-072X-3-11 15176979 PMC436063

[B4] Scott K Abhinav K Stanton B Johnston C Turner M Ampong A Sakel M Orrell R Howard R Shaw C Leigh P Al-Chalabi A Geogrphical clustering of amyotrophic lateral sclerosis in south-east England: a population study Neuroepidemiology 2009 32 81 88 19039239 10.1159/000177032

[B5] Klassen A Kulldorff M Curriero F Geographical clustering of prostate cancer grade and stage at diagnosis, before and after adjustment for risk factors Int J Health Geogr 2005 4 1 10.1186/1476-072X-4-1 15649329 PMC546220

[B6] Kleinman K Abrams A Kulldorff M Platt R A model-adjusted space-time scan statistic with an application to syndromic surveillance Epidemiol Infect 2005 133 409 419 10.1017/S0950268804003528 15962547 PMC2870264

[B7] Perez AM Zeng D Tseng C Chen H Whedbee Z Paton D Thurmond MC A web-based system for near real-time surveillance and space-time cluster analysis of foot-and-mouth disease and other animal diseases Prev Vet Med 2009 91 39 45 10.1016/j.prevetmed.2009.05.006 19505735

[B8] Kulldorff M Athas W Feuer E Miller B Key C Evaluating cluster alarms: a space-time scan statistic and brain cancer in Los Alamos Am J Public Health 1998 88 1377 1380 10.2105/AJPH.88.9.1377 9736881 PMC1509064

[B9] Lawson AB Kleinman K Spatial and syndromatic surveillance for public health 2005 Chichester, West Sussex, Hoboken, NJ: J. Wiley

[B10] Edge VL Pollari F Ng LK Michel P McEwen SA Wilson JB Jerrett M Sockett PN Martin SW Syndromic surveillance of *Norovirus*using over-the-counter sales of medications related to gastrointestinal illness Can J Infect Dis Med Microbiol 2006 17 235 241 18382634 10.1155/2006/958191 PMC2095074

[B11] Hope K Durrheim DN Muscatello D Merritt T Zheng W Massey P Cashman P Eastwood K Identifying pneumonia outbreaks of public health importance: can emergency department data assist in earlier identification? Aust N Z J Public Health 2008 32 361 364 10.1111/j.1753-6405.2008.00255.x 18782400

[B12] van Dijk A McGuinness D Rolland E Moore KM Can telehealth Ontario respiratory call volume be used as a proxy for emergency department respiratory visit surveillance by public health? CJEM 2008 10 18 24 18226314 10.1017/s1481803500009969

[B13] Vourc’h G Bridges VE Gibbens J De Groot BD McIntyre L Poland R Barnouin J Detecting emerging diseases in farm animals through clinical observations Emerg Infect Dis 2006 12 204 210 10.3201/eid1202.050498 16494743 PMC3293432

[B14] Van Metre DC Barkey DQ Salman MD Morley PS Development of a syndromic surveillance system for detection of disease among livestock entering an auction market JAVMA 2009 234 658 664 10.2460/javma.234.5.658 19250046

[B15] Vilas VJDR Bohning D Kuhnert R A comparison of the active surveillance of scrapie in the European Union Vet Res 2008 39 37 52 10.1051/vetres:2008014 18307969

[B16] Weber WD Development of an animal health monitoring system based on slaughter condemnation data 2009 Miami: Proceedings of the Eighth International Society for Disease Surveillance Conference 3 4 December

[B17] Alton GD Pearl DL Bateman KG McNab WB Berke O Factors associated with whole carcass condemnation rates in provincially-inspected abattoirs in Ontario 2001–2007: implications for food animal syndromic surveillance BMC Vet Res 2010 6 42 10.1186/1746-6148-6-42 20704738 PMC2933697

[B18] Kulldorff M Heffernan R Hartman J Assunção R Mostashari F A space-time permutation scan statistic for disease outbreak detection PLoS Med 2005 2 216 224 10.1371/journal.pmed.0020216 PMC548793 15719066

[B19] Alton GD Pearl DL Bateman KG McNab WB Berke O Suitability of portion condemnations at Ontario provincially-inspected abbatoirs for food animal syndromic surveillance BMC Vet Res 2012 8 88 10.1186/1746-6148-8-88 22726722 PMC3543186

[B20] Huang L Tiwari R Zou Z Kulldorff M Feuer E Weighted normal spatial scan statistic for heterogeneous population data J Am Stat Assoc 2009 104 487 886 898 10.1198/jasa.2009.ap07613

